# Research on Clamping Force Control of CVT for Electric Vehicles Based on Slip Characteristics

**DOI:** 10.3390/s22062131

**Published:** 2022-03-09

**Authors:** Bing Fu, Taiping Zhu, Jingang Liu, Xiaolan Hu

**Affiliations:** 1 School of Mechanical Engineering, Xiangtan University, Yuhu District, Xiangtan 411105, China; fubing@xtu.edu.cn (B.F.); 201921001814@smail.xtu.edu.cn (T.Z.); 2 Zhuzhou Gear Co., Ltd., Tianyuan District, Zhuzhou 412000, China; huxiaolan@hnu.edu.cn; 3 State Key Laboratory of Advanced Design and Manufacturing for Vehicle Body, Hunan University, Yuelu District, Changsha 410082, China

**Keywords:** continuously variable transmission, clamping force, efficiency, slip, fuzzy control, electric vehicle

## Abstract

The low mechanical efficiency of metal belt’s continuously variable transmission (CVT) limits its application in new energy vehicles. To further improve CVT efficiency and reduce the energy consumption of electric vehicles (EVs) with CVT, this paper proposes a pure electric CVT configuration and a clamping force control strategy. The slip characteristics of CVT are obtained through a bench test, the dynamic model of CVT slip is established, and a clamping force fuzzy control strategy is designed. The strategy is studied by simulation under extreme conditions and standard driving cycles. The simulation results show that the proposed clamping force control strategy has good adaptability. Under extreme conditions, this strategy can ensure that CVT does not undergo macro slip, while reducing the clamping force by 12.86–21.65%. Energy consumption per 100 km is 14.90 kWh in NEDC, which is 6.67% lower compared with the traditional strategy. CVT average efficiency and average transmission efficiency increased by 3.71% and 6.40%. The research results demonstrate that adjusting the CVT clamping force through fuzzy control based on the slip rate can improve the CVT efficiency and energy economy of EVs, which provides a certain reference for CVT clamping force control strategy development and the application of CVT on EVs.

## 1. Introduction

With the intensifying of the energy crisis and environmental pollution, many government departments have formulated increasingly strict regulations to limit vehicle energy consumption and pollutant emissions [1]. EVs are considered to be a suitable substitute for traditional vehicles because of the environmental protection, low noise, and small operating costs [2,3], which have received acute attention from automakers and consumers.

Nowadays, single-speed transmissions are used in most mass-produced EVs to save costs, such as the Volkswagen e-Golf, Nissan Leaf, and others [4]. However, since the ability to adjust the motor is very limited, the single-speed transmission sacrifices some energy economy [5]. To further improve the overall efficiency, experts and scholars have begun to study the application of multi-speed transmissions to EVs. Several studies have shown that two-speed and other multi-speed transmissions can improve the transmission efficiency of EVs, thereby enhancing vehicle dynamics [6,7,8,9]. Some scholars developed novel multi-speed transmissions for EVs to improve vehicle energy economy [10,11,12]. Additionally, the shift strategy of multi-speed transmission has also become the focus of research, and a variety of shift strategies for multi-speed transmission have been proposed in the literature [13,14,15]. Corresponding controllers have been developed to enhance vehicle efficiency and improve driving comfort. As can be seen from the above documents, multi-speed has become an inevitable trend for transmission for EVs. Some developed EVs have recently begun to apply multi-speed transmissions, such as BMW i8 and Volvo XC90 [16].

The CVT can realize the continuous change of ratio within a certain speed ratio range. The characteristics of continuously variable ratio can adjust the motor operating point more flexibly, improving vehicle energy economy [17]. The literature [18,19] compares CVT and multi-speed transmissions through simulation. The results show that CVT is the most potential and ideal transmission for EVs. However, the study did not consider the effect of CVT efficiency, which resulted in a low energy-saving effect. Ruan et al. compared the impact of different transmissions on the performance and cost of EVs through simulation. EVs with CVT show lower energy consumption and stronger power, while CVT can save customers’ money from a long-term perspective [4,20]. Research [21] compared CVT and single-speed transmission through experiments and found that CVT brings a 4.3% reduction in energy consumption for EVs. In addition, some scholars design CVT speed ratio control strategies to improve the transmission system efficiency for EVs [17,22]. The existing research on electric CVT focuses on the qualitative comparative analysis of different transmissions, few pieces of literature conduct in-depth research on the efficiency of CVT for EVs. However, the CVT efficiency is between 75% and 90% depending on the work conditions [23]. The impact of CVT efficiency on the energy consumption of EVs cannot be ignored, which is necessary to conduct relevant research.

The slip characteristic is an inherent characteristic of the metal belt CVT. Proper slip can improve the CVT efficiency, while excessive slip would cause damage to the CVT. How to make the CVT work in the high-efficiency slip range has always been the focus of CVT research. In recent years, more and more scholars have conducted research on slip control methods. Asayama et al. and Kobyyashi et al. conducted research from the perspective of theory and experimentation by analyzing the relationship between clamping force and slip rate [24,25]. Kim et al. investigated the metal V-belt behavior of CVT both analytically and experimentally. They obtained the ratio-torque load-axial force relationship and found that the torque transfer capability of the driven side determines the overall slip [26]. Nishizawa et al. studied the friction characteristics between the CVT metal belt and the pulley and proposed a friction model and obtained the power transmission state near the macro slip limit [27]. Tarutani et al. established an effective model of CVT load distribution, analyzed the influence of slip velocity on power transmission, and verified the validity of the calculation results through experiments [28]. Bonsen et al. and Klaassen et al. studied the slip characteristics of metal belts by combining theory with experiments. They propose a CVT slip model and develop corresponding slip control controllers. The research results show that controlling the slip can improve CVT efficiency [29,30,31]. In the literature [32,33,34,35], various controllers such as the adaptive PI controller and LQR controller are designed to improve the effect of slip control. Simulation results show that the designed controller can improve the efficiency of CVT without losing robustness. Additionally, Zhu et al. established a vehicle driving state identification method and analyzed the feasibility of slip control in unsteady states. They proposed a clamping force control method in the unsteady states and verified the method through experiments [36]. Ji et al. studied the power transfer characteristics under different slip regions under external vibration conditions. The results indicate that there are differences in the external vibration transfer characteristics between the micro-slip region and the macro-slip region [37]. The mentioned documents mostly study CVT slip characteristics separately from experiments or combine CVT slip control with traditional vehicles, and few studies combine EVs with CVT slip control for efficiency optimization research.

This paper presents a pure electric CVT configuration, and a clamping force fuzzy control strategy based on slip characteristics. CVT slip dynamic model and vehicle simulation model are established. The feasibility of the proposed clamping force control strategy and the superiority over the traditional clamping force control strategy are verified by simulation. The structure of this article is as follows: Section 2 introduces the configuration of pure electric CVT. Section 3 describes the slip principle of belt CVT. Section 4 accomplishes CVT slip dynamic model and vehicle model modeling. Section 5 performs the slip characteristic test. Section 6 describes the CVT clamping force control strategy. Section 7 analyzes and discusses the simulation results. Finally, the conclusion is summarized in Section 8.

## 2. Configuration of Electric Vehicle with CVT

**Figure 1:**
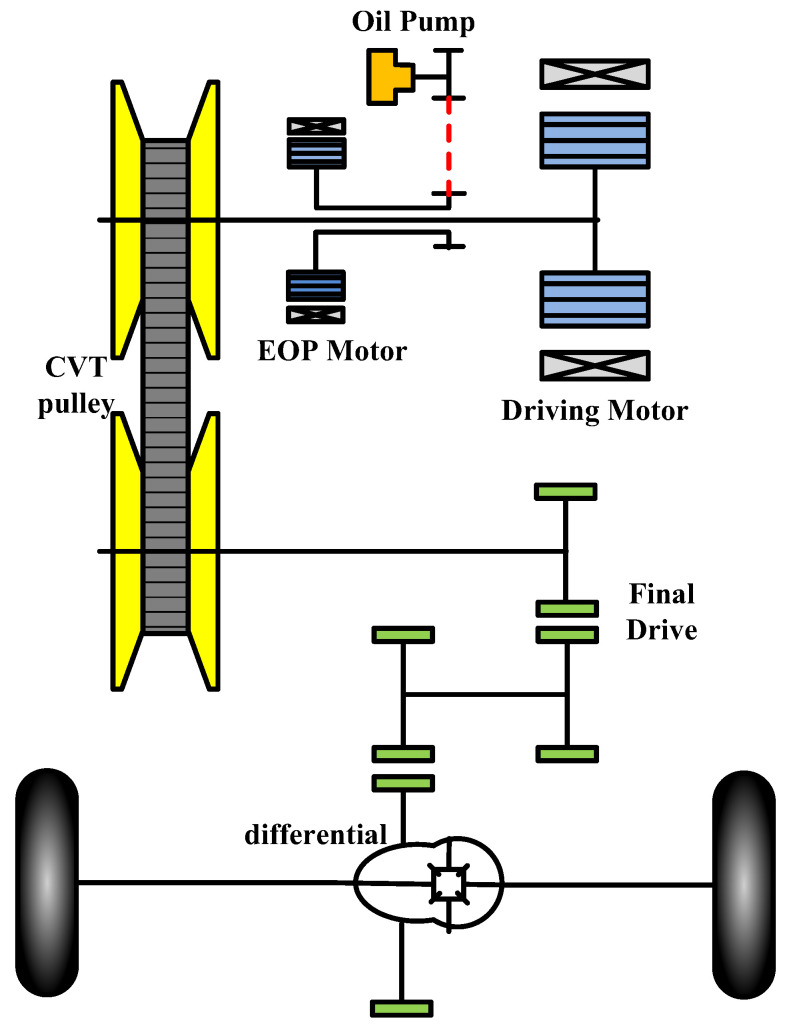
The structure of EV proposed.

The configuration of the pure electric CVT proposed in this paper is shown in Figure 1. The structure is similar to that of the traditional CVT structure, but the hydraulic torque converter and clutch are eliminated according to functional requirements. The hydraulic system employs the electric oil pump (EOP) as the power source, which can realize the independent drive of the oil pump.

**Figure 2:**
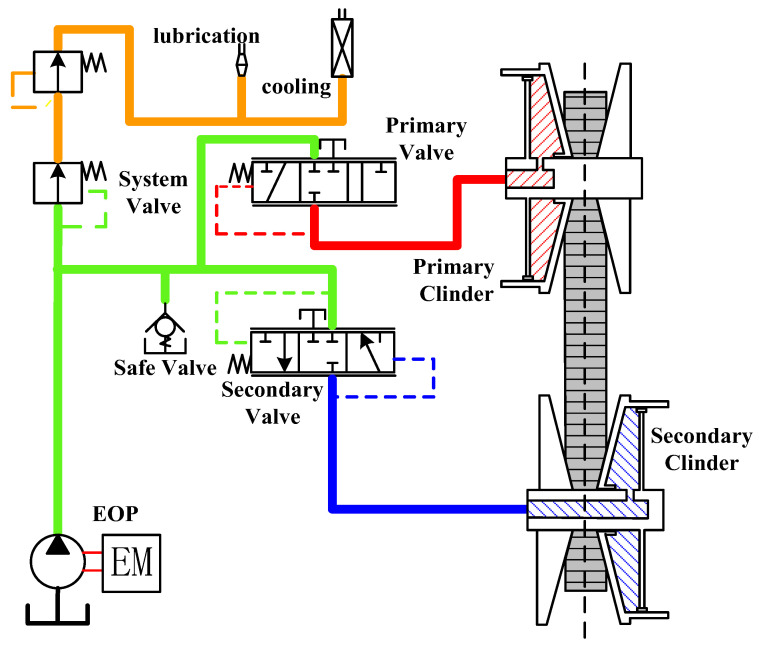
Schematic diagram of hydraulic system.

Figure 2 displays the hydraulic system construction for the pure electric CVT. The oil pump is driven by a separate motor rather than coupled with the speed of the drive motor. The oil circuit can be divided into three parts according to the oil pressure. The first part provides pressure on the secondary side, which is used to control the clamping force of the CVT and ensure the torque transmission. The second part supplies pressure for CVT speed ratio adjustment. The third part is used to cool and lubricate the components of the transmission system.

## 3. Belt CVT Slip Principle

The metal belt CVT transmission mode is friction transmission. The clamping forces of the hydraulic cylinders exert on the primary and secondary pulleys, and the metal belt transmits torque from the primary side to the secondary side through friction. To ensure the reliability of power transmission, the following formula is generally used to calculate the clamping force of the secondary pulley:

(1) $$F_{se}=\frac{S_{fr}T_{pm}cos\alpha }{2\mu R_{se}}$$

where *F_se_* is the secondary clamping force, *S_fr_* is the safety factor, *T_pm_* is the transmitted torque, *α* is the cone angle of the pulley, *μ* is the friction coefficient between metal belt and cone plate, and *R_se_* is the effective radius of the secondary pulley.

**Figure 3:**
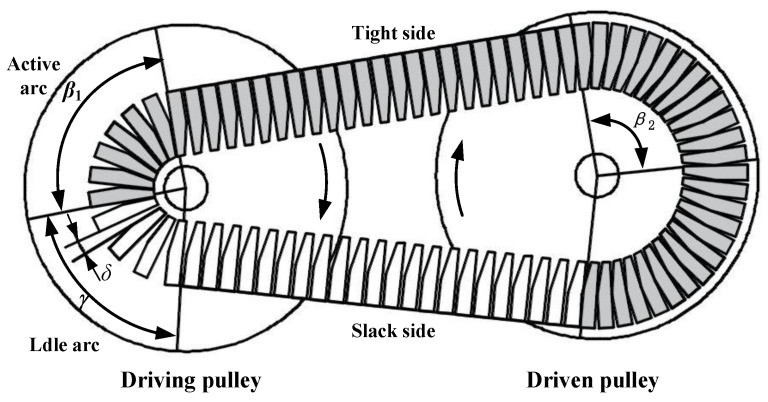
Principle of metal belt slip (low-speed ratio).

In the process of metal belt transmission, the metal belt can be divided into two states in the pulley envelope: active arc and idle arc, and the metal belt is divided into the tight side and slack side, as shown in Figure 3. 

There is a squeezing force between the metal sheets at the tight side, while the slack side has no squeezing force. Due to the gap inconsistency between the tight side and slack side, the pulley will slip during transmission [25]. The slip rate s can be expressed as:

(2) $$s=1-\frac{i_{r}}{i_{o}}$$

(3) $$i_{o}=\frac{n_{pm}}{n_{se}}$$

where *i_r_* is the speed ratio without load, and *i_o_* is the actual speed ratio, *n_pm_* is the primary pulley speed, and *n_se_* is the secondary pulley speed.

**Figure 4:**
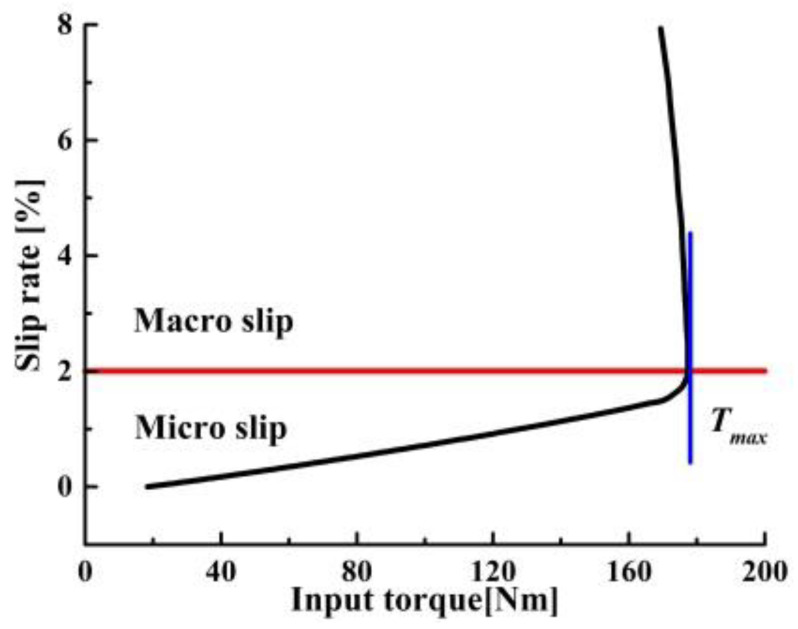
Schematic diagram of slip region.

When the clamping force is constant, DROGEN et al. divided the CVT slip into the macro slip and micro slip under the condition of certain clamping force [38], as shown in Figure 4. Macro slip is commonly referred to as metal belt slip. It could cause rapid wear of the steel belt and affect the transmission, which should be avoided as much as possible.

## 4. Vehicle and CVT Dynamic Models

### 4.1. Vehicle Dynamics Model

The vehicle is subjected to the following resistances during driving: rolling resistance *F_f_*, air resistance *F_w_*, climbing resistance *F_i_*, and acceleration resistance *F_j_*. The driving force of the vehicle is balanced with the resultant force of these forces. Therefore, the vehicle dynamics model can be expressed as:

(4) $$F_{d}=F_{f}+F_{w}+F_{i}+F_{j}=mgf\cos\phi +\frac{C_{D}Av}{21.15}+mg\sin\phi +\varepsilon m\frac{dv}{dt}$$

where *F_d_* is the vehicle driving force, *m* is the mass of the vehicle, *g* is the acceleration of gravity, *f* is the wheel rolling resistance coefficient, *C_D_* is the air resistance coefficient, *A* is the frontal area, *v* is vehicle speed, ϕ is the road gradient, and *ε* is the rotation mass conversion coefficient.

### 4.2. CVT Slip Dynamic Model

To reduce the difficulty of constructing the CVT slip model, several assumptions are made as follows. 1. Ignore the elasticity of the transmission shaft and the vibration during transmission; 2. assume that there is no gap between the shaft and the cone plate; 3. ignore some system damping; 4. ignore the influence of the hydraulic system on CVT transmission characteristics; and 5. use the geometric speed ratio *i_g_* to replace the no-load speed ratio *i_r_*, because the geometric speed ratio can be calculated from the displacement sensor data.

(5) $$i_{g}=\frac{R_{se}}{R_{pm}}$$

where *R_pm_* is the effective radius of the primary pulley when the speed ratio is *i_g_*.

**Figure 5:**
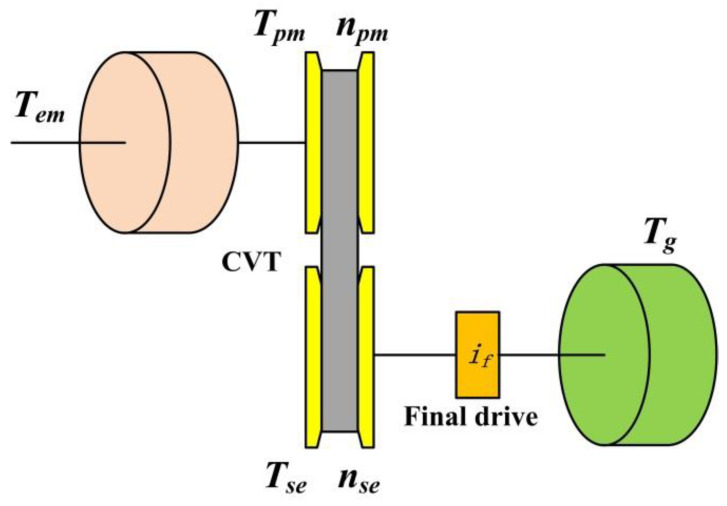
Diagram of transmission system.

The CVT transmission system is shown in Figure 5. The transmission model reflects the relationship between input and output and is appropriately simplified when modeling. The specific mathematical model is as follows:

(6) $$I_{p}\frac{dn_{pm}}{dt}=T_{em}-T_{pm}$$

(7) $$n_{pm}=i_{cvt}n_{se}$$

(8) $$T_{se}=T_{pm}i_{cvt}\eta_{b}$$

(9) $$\frac{dn_{pm}}{dt}=i_{cvt}\frac{dn_{se}}{dt}+n_{s}\frac{di_{cvt}}{dt}$$

where *I_p_* and *I_s_* are the input and output moments of inertia of the CVT, *T_em_* is the motor torque, *T_q_* is the torque of the wheel drive shaft, *T_se_* are the torque secondary pulley, *i_f_* is the speed ratio of final drive, *i_cvt_* is the speed ratio of CVT, and *η_b_* is the efficiency of the metal belt.

Combining Equations (2), (3) and (5):

(10) $$\overset{\cdot }{s}=\frac{{\overset{\cdot }{n}}_{se}n_{pm}-n_{se}{\overset{\cdot }{n}}_{pm}}{{n_{se}}^{2}i_{o}}$$

Combining Equations (5)–(10) can obtain the mathematical CVT slip model:

(11) $$\overset{\cdot }{s}=\frac{\left(1-s\right)}{n_{se}}(\frac{T_{L}}{I_{se}}-\frac{2F_{se}R_{se}\mu }{I_{\mathrm{se}}cos\alpha })+\frac{1}{n_{se}i_{o}}(T_{em}-\frac{2F_{se}R_{se}\mu }{i_{o}I_{pm}cos\alpha })$$

### 4.3. EOP Model

The EOP was selected as the power source for the hydraulic system. The oil pump is driven by a separate oil pump motor and is not affected by the drive motor. The relationship between the oil pump torque, flow and displacement can be calculated by the following Equations:

(12) $$T_{p}=\frac{P_{p}V_{p}}{2\pi \eta_{jy}}$$

(13) $$Q_{p}=V_{p}n_{p}\eta_{rx}$$

(14) $$P_{p}=\frac{P_{sy}V_{p}}{60\eta_{pt}}$$

where *T_p_* is oil pump torque, *P_p_* is the pressure difference of the oil pump, *V_p_* is oil pump displacement, *Q_p_* is the hydraulic oil flow rate, *n_p_* is oil pump speed, *P_sy_* is system pressure, *η_jy_* is oil pump hydro-mechanical efficiency, *η_rx_* is oil pump volumetric efficiency, and *η_pt_* is the overall efficiency of the oil pump.

Since this paper focuses on the study of CVT clamping force control, the EOP control strategy is not considered in this article. It is assumed that the transmission control unit (TCU) can automatically adjust the EOP speed according to the system flow demand, realizing the active control of the system flow.

### 4.4. Model Integration

**Figure 6:**
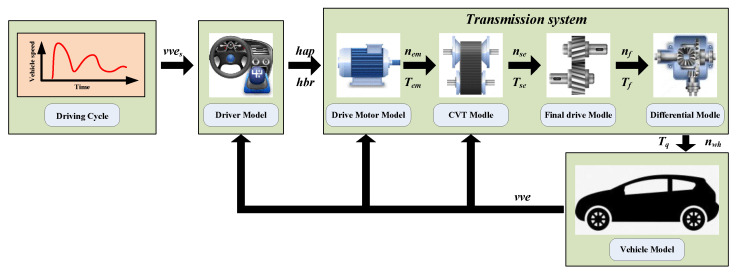
Simulation system structure.

The simulation system is presented in Figure 6, including the driver model, vehicle model, motor model, and the CVT slip dynamic model. The simulation system takes the target vehicle speed *vve_s_* as the input and the driver model outputs the accelerator opening *h_ap_* and brake pedal opening *h_br_*. The transmission system transfers the wheel speed *n_wh_* and torque *T_q_* to the vehicle model to perform the simulation.

## 5. CVT Slip-Efficiency Characteristic Test

**Figure 7:**
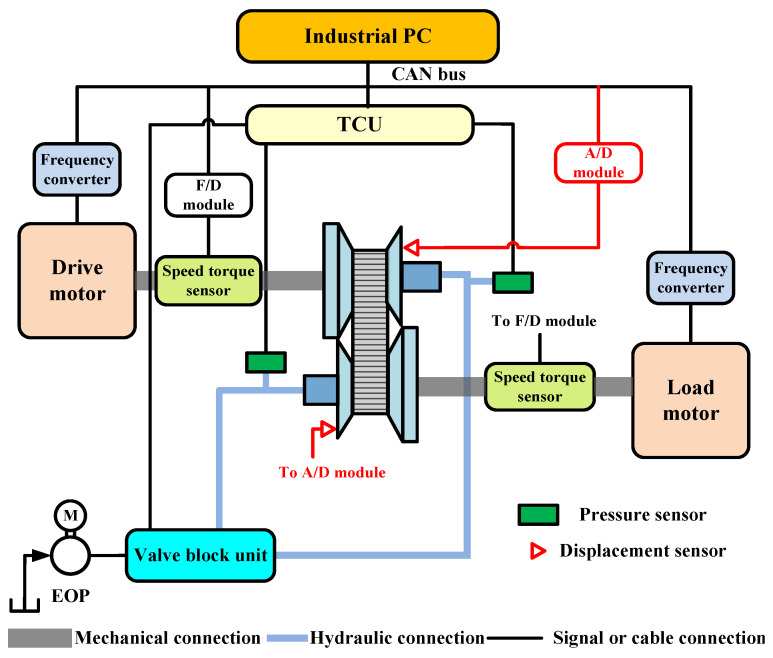
Schematic diagram of test scheme.

**Figure 8:**
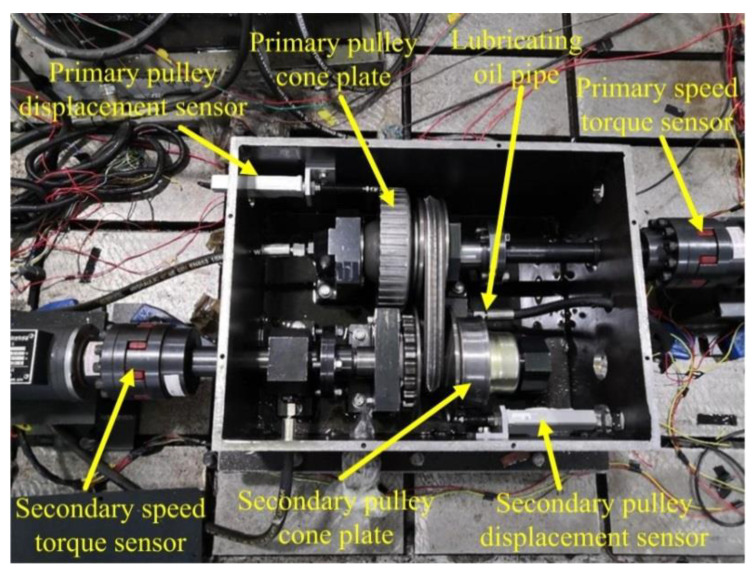
CVT slip characteristic test bench.

Within a certain slip rate, the friction coefficient and the mechanical efficiency of CVT show the same trend-first increase to the peak and then decrease [27,28]. Thus, the efficiency can be improved by reasonably controlling CVT slip. To design a clamping force control strategy, it is necessary to obtain accurate CVT slip characteristics and their relationship with efficiency. Therefore, a slip characteristic test bench was built. Figure 7 is the schematic diagram of the test bench, and the actual slip test bench is shown in Figure 8. The test device is mainly composed of drive motor, load motor, CVT, TCU, various sensors, and data acquisition system. The drive motor shaft is directly connected to the primary pulley, and the load motor simulates the road load. Considering the influence of oil temperature on the efficiency of CVT, the oil temperature was stabilized at 90 ± 5 °C during the experiment.

**Figure 9:**
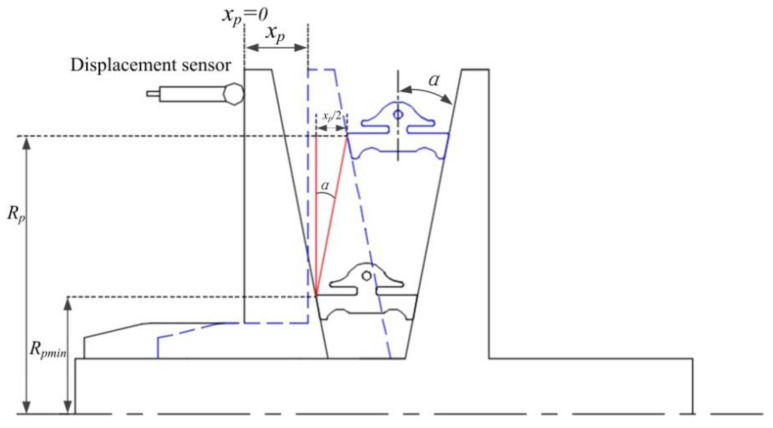
Primary pulley radius calculation principle.

The experimental transmission is a Chinese company self-developed CVT. CVT-related parameters are given in Table 1. The slip rate is defined by the geometric speed ratio and the actual speed ratio, as shown in Equation (2). During the experiment, the actual speed ratio is calculated by data from the primary and secondary speed sensors. The geometric speed ratio is the ratio of the radius of the primary and secondary pulley, which cannot be obtained directly. It can be calculated indirectly through the axial displacement of primary and secondary cone plates measured by the displacement sensors. Here, the calculation of the radius of the primary pulley is taken as an example, as shown in Figure 9. The specifications and parameters of various sensors and other components are shown in Table 2. The actual radius of the primary and secondary pulley can be expressed as:

(15) $$R_{pm}=R_{pmin}+\frac{x_{p}}{2\tan\alpha }$$

(16) $$R_{se}=R_{smin}+\frac{x_{s}}{2\tan\alpha }$$

where *x_p_* and *x_s_* are the displacement of the movable cone plate of the primary and secondary pulleys. *R_pmin_* and *R_smin_* are the radius of the primary and secondary pulleys when *x_p_* = 0 and *x_s_* = 0.

Based on the consideration of the typical working conditions of CVT, the efficiency characteristic tests under different speed ratios, input speeds, and torques were carried out, respectively. The setting of working conditions is shown in Table 3. In the test, the speed and torque of the drive motor and the load motor are measured by the speed-torque sensors, and the CVT efficiency is calculated by the following formula:

(17) $$\eta_{cvt}=\frac{T_{e2}n_{e2}}{T_{e1}n_{e1}}$$

where *T_e_*_1_ and *T_e_*_2_ are the torque of the drive motor and load motor, and *n_e_*_1_ and *n_e_*_2_ are the speed of the drive motor and load motor.

**Figure 10:**
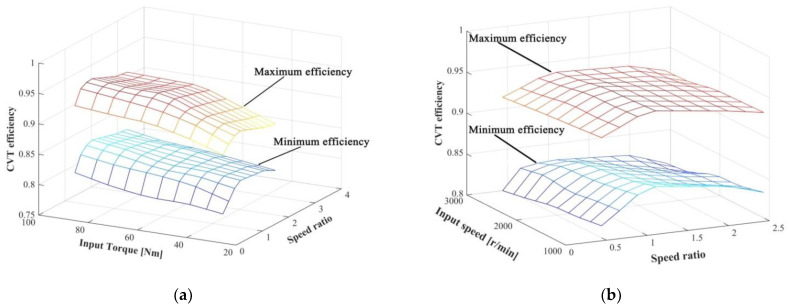
The relationship between CVT extreme efficiency and speed and torque: (**a**) the relationship between CVT efficiency and input torque and speed ratio (*ω_pm_* = 2000 r/min); (**b**) the relationship between CVT efficiency and input speed and speed ratio (*T_pm_* = 60 Nm).

Figure 10 shows the minimum and maximum efficiency of the transmission mechanism under different test conditions. It can be seen from the Figure that there is a margin of 5–10% between the minimum and maximum efficiency within the slip region. This indicates that the slip has a significant impact on CVT efficiency, and the CVT efficiency can be improved by controlling the slip rate.

**Figure 11:**
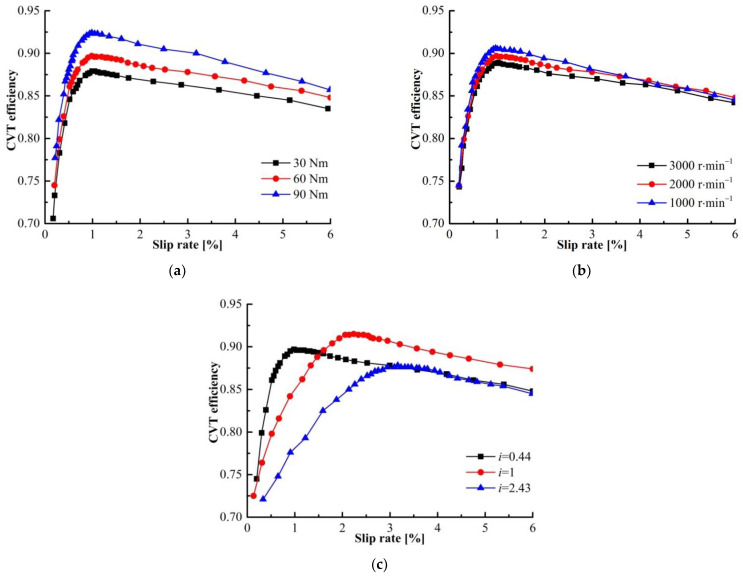
CVT slip-efficiency characteristics: (**a**) slip-efficiency characteristics under different input torque; (**b**) slip-efficiency characteristics under different input speed; (**c**) slip-efficiency characteristics under different speed ratio.

Figure 11 shows CVT slip-efficiency characteristics under different test conditions. Figure 11a–c shows similar trends in the CVT efficiency, but the characteristics of the optimal slip rate are not completely the same. The CVT slip-characteristic is more sensitive to the speed ratio, and the slip rate corresponding to the optimal efficiency at different speed ratios is quite different. The input speed has little effect on the CVT slip-efficiency characteristics, the variation of the CVT efficiency is within 2%. It is worth noting that the slip rate under the optimal efficiency hardly changes with the input speed and torque in Figure 11a,b. Therefore, the optimal target slip rate can be defined by the speed ratio:

(18) $$s_{tar}=f(i_{cvt})$$

## 6. Clamping Force Control Strategy

From the principal analysis in Section 3 and the modeling process in Section 4, it can be seen that the clamping force is significant to the transmission performance of the CVT. The torque that the metal belt can transmit is limited by the clamping force. When the input torque is lower than the maximum torque, the macro slip will not occur in the CVT. If the input torque is excessive, it will cause the metal belt to slide and affect the power transmission.

### 6.1. Traditional Clamping Force Control Strategy

**Figure 12:**
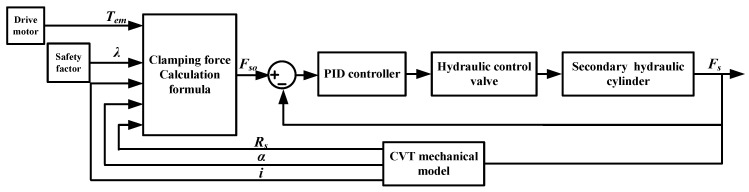
Traditional clamping force control strategy.

In the traditional clamping force control strategy, the safety factor is set to a larger fixed value to avoid CVT macro slip. In some extreme conditions, the safety factor may be set higher. Setting the clamping force in this way can effectively prevent the metal belt from slip. However, excessive clamping force may result in lower CVT efficiency and higher hydraulic system loss. The traditional CVT clamping force control strategy is shown in Figure 12.

### 6.2. Clamping Force Fuzzy Control Strategy Based on the Slip Rate

The traditional clamping force control strategy avoids macro slip by setting a large safety factor, which sacrifices some CVT efficiency. As can be seen from Figure 11a, there is a maximum efficiency point for each speed ratio under a certain input condition. To sustain a high mechanical efficiency of the CVT, it is vital to keep the slip rate near the optimal efficiency.

**Figure 13:**
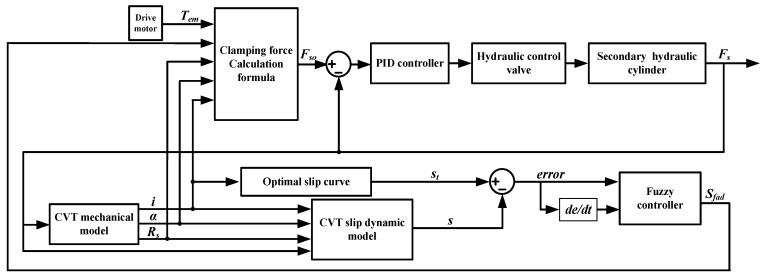
Clamping force fuzzy control strategy based on slip rate.

To ensure the slip rate is near the optimal slip rate, this paper proposes a clamping force fuzzy control strategy based on slip rate. This strategy adopts dual closed-loop control, as shown in Figure 13. The inner loop adopts PID control. The controller controls the hydraulic valve and cylinder to adjust the clamping force of the secondary pulley. The fuzzy controller of the outer loop adjusts the dynamic safety factor based on the slip state to correct the target clamping force, facilitating the slip rate to approach the optimal slip rate.

**Figure 14:**
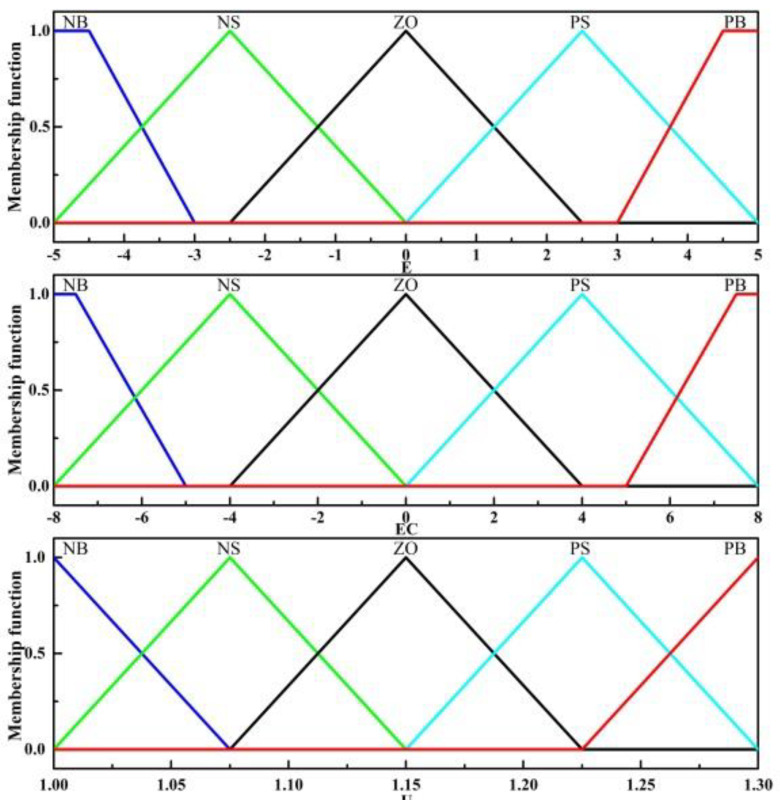
Input and output membership function.

The fuzzy control system is a system with two inputs and one output. The inputs of the controller are the error of slip rate and changing rate of error. Their domains are [−5, −4, −3, −2, −1, 0, 1, 2, 3, 4, 5] and [−8, −6, −4, −2, 0, 2, 4, 6, 8]. The domain of the system output is [1, 1.05, 1.10, 1.20, 1.25, 1.30]. The membership function in the middle part of the input adopts the trigonometric function, and the trapezoidal function is adopted at the two edges. The output membership functions are all trigonometric functions. The fuzzy language variables are the following: NB, NS, ZO, PS, PB. Figure 14 shows the input and output membership functions of the fuzzy system. Table 4 shows the fuzzy control rules.

## 7. Simulation and Result Analysis

### 7.1. Simulation Parameter Setting

The simulation parameters are exhibited in Table 5. Subsequent simulations in this Section will be on the basis of the model built in the previous article and the data in the table. The feasibility of the proposed clamping force control strategy and the energy consumption benefits it can bring are investigated by simulation, the reciprocal of the actual safety factor 1/*S_fr_* is defined as:

(19) $$1/S_{fr}=\frac{F_{sen}}{F_{ser}}$$

where *F_sen_* is required clamping force, *F_ser_* is actual clamping force.

It is generally considered that when the reciprocal of the safety factor is lower than the critical value, the macro slip will not occur in CVT.

### 7.2. Extreme Conditions Simulation

**Figure 15:**
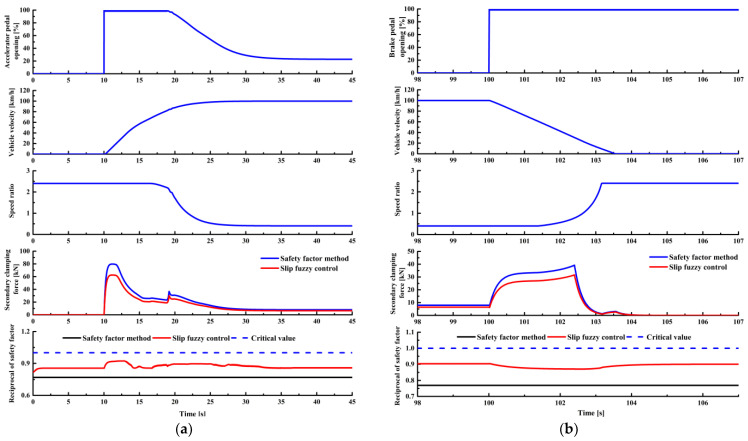
Simulation results of acceleration and braking conditions: (**a**) full throttle acceleration conditions; (**b**) emergency braking conditions.

Figure 15a,b show the simulation results of acceleration and emergency braking conditions. It can be seen that the 1/*S_fr_* of the proposed control strategy in the two working conditions is lower than the critical value. This means that there is no risk of macro slip in the metal belt during transmission. Compared with the traditional safety factor strategy, the clamping force slip fuzzy control strategy can reduce the CVT clamping force. In the two conditions, the clamping force can be reduced by a maximum of 19.49% and 21.65%, respectively. This is because the proposed control strategy can adjust the target clamping force according to the current slip state of the CVT, which leads to a reduction in clamping force. Due to the limited power of the motor, the output torque of the motor drops at about 13 s to ensure the output speed, so the clamping force presents a process of fluctuations.

**Figure 16:**
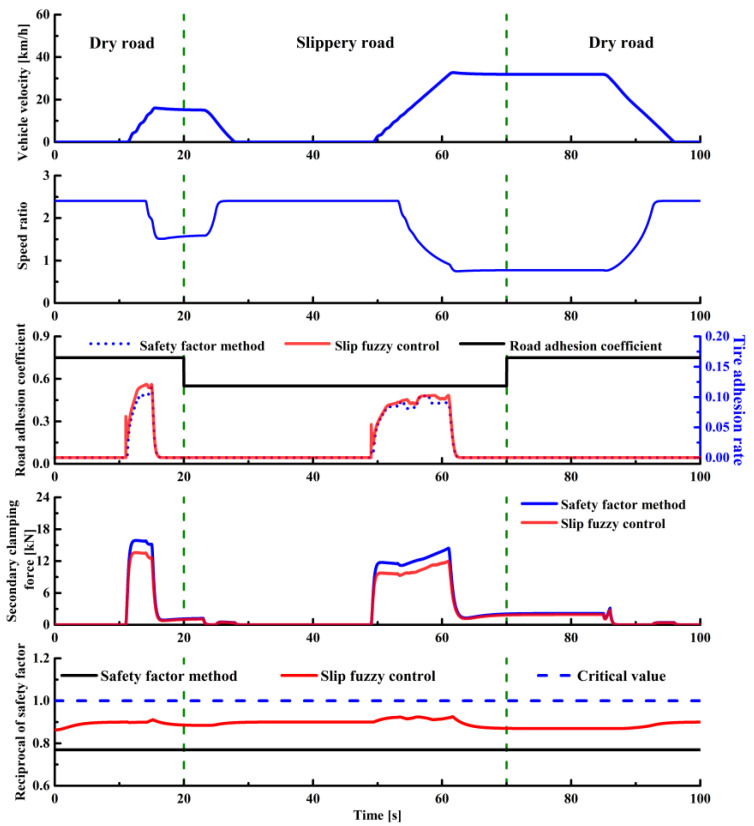
Road adhesion coefficient abrupt change condition.

The simulation results of the road adhesion coefficient abrupt change condition are presented in Figure 16. The vehicle enters the slippery road from a dry road at 20 s and then drives to a dry road again at 70 s. When the road adhesion coefficient changes, the drive tire adhesion rate is consistently lower than the road adhesion coefficient, so the drive wheels will not slip. At the same time, 1/*S_fr_* is lower than the critical value, and there is no macro slip in CVT. Additionally, the clamping force fuzzy control strategy reduces the clamping force to a certain extent.

Synthesizing the simulation results of the three working conditions, it is clear that the clamping force fuzzy control strategy based on slip has good adaptability. The proposed strategy can still ensure the metal belt does not slip under extreme conditions; thus, this strategy is feasible for clamping force control. In the next section, simulations will be conducted under standard driving cycles, and explore the impact of the proposed strategy on vehicle energy consumption and transmission efficiency.

### 7.3. Simulation under Standard Driving Cycles

**Figure 17:**
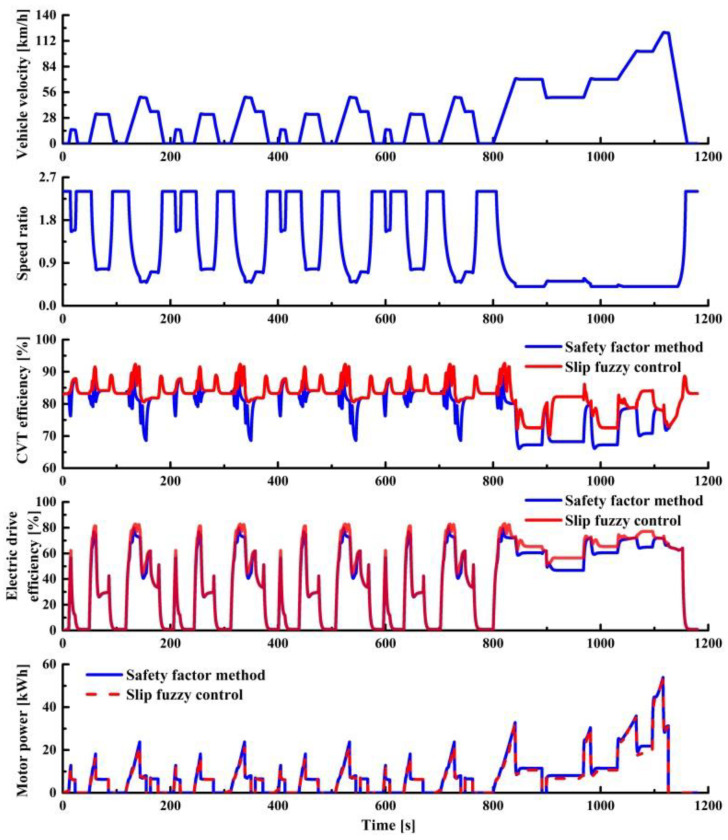
Simulation results in NEDC driving cycle.

Figure 17 shows the vehicle speed, speed ratio, and transmission efficiency with different clamping force control strategies under NEDC driving cycle. It can be seen from the Figure that the clamping force fuzzy control strategy based on slip rate improves the CVT mechanical efficiency and electric drive efficiency. The average efficiency of CVT and the electric drive efficiency increased by 3.41% and 6.40%, respectively. These results could be attributed to the timely adjustment of clamping force. When the vehicle is driving, the fuzzy controller adjusts the clamping force according to the slip state of the CVT, leading to an increase in efficiency. The improvement of transmission efficiency and reduction in motor power bring an improvement in vehicle energy consumption. Table 6 shows the simulation results with different clamping force control strategies under NEDC. The vehicle energy consumption with clamping force fuzzy control strategy based on slip rate is 14.90 kWh, which is reduced by 6.67% compared to the traditional clamping force control strategy.

**Figure 18:**
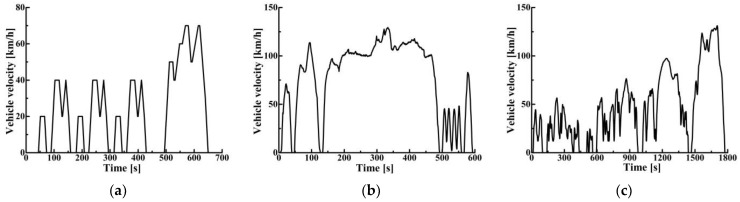
Vehicle velocity profiles of different driving cycles: (**a**) 1015 driving cycle; (**b**) US06 driving cycle; (**c**) WLTC driving cycle.

In order to further study the energy-saving effect of this strategy, simulations were carried out under 1015, US06 and WLTC driving cycles. The average vehicle speeds in the above driving cycles are 22.7 km/h, 77.9 km/h and 46.0 km/h. The 1015, NEDC, and US06 represent typical low-speed, medium-speed, and high-speed driving cycles, respectively. The WLTC is a driving cycle where the vehicle speed changes drastically. The above driving cycles can comprehensively reflect the comprehensive characteristics of vehicle driving. The speed of various driving cycles is shown in Figure 18.

The energy consumption and CVT efficiency with different strategies under various driving cycles are given in Table 7 and Table 8. Simulation results demonstrate that the clamping force fuzzy control strategy based on slip rate can reduce vehicle energy consumption and improve CVT efficiency under the tested driving cycles. This control strategy reduces the energy consumption by 6.21% at the maximum and 4.53% at the minimum, and CVT efficiency increased by 1.78% to 3.99%. The improvement of energy consumption and CVT efficiency is most remarkable in US06. An explanation for this might be that US06 is a high-speed working condition. The large torque transmission requires a larger clamping force, which makes the CVT efficiency lower with the traditional strategy. The smallest energy consumption benefit occurs in WLTC. These results further support the idea that the proposed clamping force control strategy has wonderful adaptability and can improve the vehicle economy.

**Figure 19:**
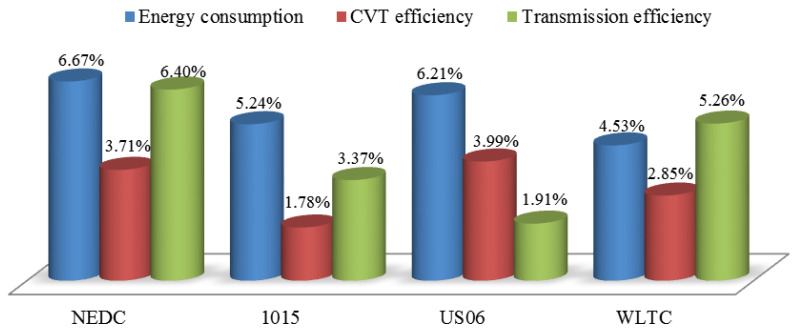
The improvement of various indicators under different driving cycles.

The improvement rate of each index under different driving cycles is shown in Figure 19. The transmission efficiency here refers to the total efficiency of the motor and CVT. It can be seen from the simulation results that the clamping force fuzzy control strategy based on slip rate can bring positive efficiency and energy consumption benefits.

## 8. Conclusions

To improve the mechanical efficiency of CVT for EVs, an electric CVT configuration and a clamping force fuzzy control strategy based on slip rate are proposed. The strategy adopts dual closed-loop control. The fuzzy controller outputs dynamic safety factors to adjust the clamping force based on the slip state of the CVT. The vehicle dynamics model and the CVT slip dynamic model are established, and the proposed strategy is studied through simulation.

To design an appropriate clamping force control strategy, bench tests were carried out to get CVT slip-efficiency characteristics. The experimental results show that the CVT slip-efficiency characteristics show similar trends under different input conditions. The CVT efficiency is most sensitive to the speed ratio, the optimal slip rate is quite different under different speed ratios. The influence of input speed on slip-performance characteristics can be neglected. In addition, different input speeds or input torques hardly affect the optimal slip position.

Simulations were performed under three extreme conditions: full-throttle acceleration conditions, emergency braking conditions, and road adhesion coefficient mutation conditions. The simulation results indicate that the proposed clamping force control strategy can ensure that the CVT does not occur macro slip. When the road adhesion coefficient changes suddenly, the driving wheels will not slip. Compared with the traditional clamping force control strategy, the proposed strategy can reduce the CVT clamping force by 12.86–21.65%.

Under the NEDC driving cycle, the energy consumption with the clamping force slip distorted control strategy is 14.90 kWh, which is 6.67% lower than the traditional clamping force control strategy. At the same time, the average efficiency of CVT and average transmission efficiency increased by 3.71% and 6.40%, respectively. Vehicle energy consumption and CVT efficiency have also been improved to varying degrees in other standard driving cycles. Therefore, the proposed strategy can provide a reference for CVT clamping force research and electric vehicle energy consumption research.

Future works will focus on verifying the feasibility of the proposed clamping force control strategy through bench tests. At the same time, the electric oil pump control strategy will be developed to further improve the CVT efficiency and vehicle energy economy.

## Figures and Tables

**Table 1:** CVT specifications and parameters.

Parameter	Value
Metal belt type	VDT—24/9/1.5/196.8
Speed ratio range	0.44–2.43
Pulley center distance	146 mm
Number of metal bands	408
Metal belt length	612 mm
Hydraulic oil type	Idemitsu EX1
EOP type	Rotating vane pump

**Table 2:** Specifications of various sensors and other components.

Parameter	Value
Drive motor specification/rated speed/torque	ABB-QABP225M2A/3000 rpm/134 Nm
Load motor specification/rated speed/torque	ABB-QABP280S2A/3000 rpm/239 Nm
Displacement sensor specifications/measurement range/accuracy	KEYENCE-GT2-S5/0–32 mm/±2 um
Torque sensor specifications/measurement range/accuracy	Sanjing-JN338-A200/20–300 Nm/±0.05% FS
Speed sensor specifications/measurement range/accuracy	Sanjing-JN338-A200/0–5000 rpm/±2 rpm
Pressure sensor specifications/measurement range/accuracy	Nexo-PA2000/0–6 Mpa/2% FS

**Table 3:** Test conditions setting.

Test Conditions	Speed Ratio	Input Speed (r/min)	Input Torque (Nm)
Different speed ratio test	0.44, 0.60, 1.00, 2.00, 2.43	2000	60
Different input torque test	0.44	2000	30, 45, 60, 75, 90
Different speed input test	0.44	1000, 1500, 2000, 2500, 3000	60

**Table 4:** Fuzzy control rules.

	E	NB	NS	ZO	PS	PB
EC						
SB		PB	PB	PS	ZO	NS
NS		PB	PB	PS	NS	NS
ZO		PB	PS	ZO	NS	NB
PS		PS	PS	ZO	NS	NB
PB		PS	PS	NS	NB	NB

**Table 5:** Simulation parameters.

Parameter	Value
Vehicle mass	1505 kg
Wheel radius	0.3065 m
Frontal area	2.13 m^2^
CVT Speed ratio range	0.43–2.43
Final drive ratio	6.08
Pulley cone angle	11°
Oil pump displacement	15 mL/r
Final drive efficiency	98%
Differential efficiency	98%

**Table 6:** Comparison of efficiency and energy consumption.

	Traditional Clamping Force Control Method	Clamping Force Fuzzy Control Strategy Based on Slip Rate	Improvement
Average CVT mechanical efficiency	79.77%	82.84%	3.71%
Average electric drive efficiency	46.21%	49.37%	6.40%
Energy consumption per hundred kilometers	15.87 kWh	14.90 kWh	6.67%

**Table 7:** Energy consumption comparison under various driving cycles.

Driving Cycles	Energy Consumption (kWh)		Improvement
	Traditional Clamping Force Control Strategy	Clamping Force Fuzzy Control Strategy Based on Slip Rate	
1015	16.33	15.48	5.21%
US06	16.58	15.55	6.21%
WLTC	16.65	15.89	4.53%

**Table 8:** Comparison of CVT efficiency under various driving cycles.

Driving Cycles	CVT Efficiency		Improvement
	Traditional Clamping Force Control Strategy	Clamping Force Fuzzy Control Strategy Based on Slip Rate	
1015	83.72%	85.24%	1.78%
US06	78.67%	81.81%	3.99%
WLTC	80.46%	82.75%	2.85%

## Data Availability

Not applicable.
